# Digoxin Suppresses Tumor Malignancy through Inhibiting Multiple Src-Related Signaling Pathways in Non-Small Cell Lung Cancer

**DOI:** 10.1371/journal.pone.0123305

**Published:** 2015-05-08

**Authors:** Sheng-Yi Lin, Hsiu-Hui Chang, Yi-Hua Lai, Ching-Hsiung Lin, Min-Hsuan Chen, Gee-Chen Chang, Meng-Feng Tsai, Jeremy J. W. Chen

**Affiliations:** 1 Institute of Biomedical Sciences, National Chung Hsing University, Taichung, Taiwan; 2 Agricultural Biotechnology Center, National Chung Hsing University, Taichung, Taiwan; 3 Division of Chest Medicine, Department of Internal Medicine, Changhua Christian Hospital, Changhua, Taiwan; 4 Division of Chest Medicine, Department of Internal Medicine, Taichung Veterans General Hospital, Taichung, Taiwan; 5 Department of Molecular Biotechnology, Dayeh University, Changhua, Taiwan; H. Lee Moffitt Cancer Center & Research Institute, UNITED STATES

## Abstract

Non-small cell lung cancer is the predominant type of lung cancer, resulting in high mortality worldwide. Digoxin, a cardiac glycoside, has recently been suggested to be a novel chemotherapeutic agent. Src is an oncogene that plays an important role in cancer progression and is therefore a potential target for cancer therapy. Here, we investigated whether digoxin could suppress lung cancer progression through the inhibition of Src activity. The effects of digoxin on lung cancer cell functions were investigated using colony formation, migration and invasion assays. Western blotting and qPCR assays were used to analyze the mRNA and protein expression levels of Src and its downstream proteins, and a cell viability assay was used to measure cellular cytotoxicity effects. The results of the cell function assays revealed that digoxin inhibited the proliferation, invasion, migration, and colony formation of A549 lung cancer cells. Similar effects of digoxin were also observed in other lung cancer cell lines. Furthermore, we found that digoxin significantly suppressed Src activity and its protein expression in a dose- and time-dependent manner as well as reduced EGFR and STAT3 activity. Our data suggest that digoxin is a potential anticancer agent that may suppress lung cancer progression through inhibiting Src and the activity of related proteins.

## Introduction

Non-small cell lung cancer (NSCLC) is the predominant type of lung cancer and the leading cause of cancer deaths worldwide [1]. The low survival rate of lung cancer patients is due to tumor resistance to adjuvant chemotherapy and metastasis [2]. The metastasis of cancer cells from primary tumors is a multi-step process that occurs through blood vessels or lymphatic vessels. To date, there is no effective therapy to inhibit or control these metastatic processes.

Oncogene dependence determines the appropriate treatment of cancers with targeted therapies. NSCLCs have many driver mutations, including those in EGFR, HER2, KRAS, BRAF, PIK3CA, AKT1, MEK1, ROS1, and ALK [3]. These driver mutations affect tumor sensitivity to cancer therapies. EGFR mutations exemplify the therapeutic relevance of molecular clusters. Clinical and biological data extensively show that EGFR mutations can predict the efficacy of EGFR inhibitors, with response rates higher than 70% and prolonged progression-free survival observed in multiple studies [4,5]. However, these inhibitors, e.g., Irresa and Tarceva, are ultimately limited by the emergence of drug-resistance mutations and other putative molecular mechanisms [6,7]. Thus, identifying novel compounds that target tumor progression, including growth and metastasis, is a matter of great urgency in cancer therapy research.

The oncogene Src plays an important role in cancer progression and causes poor prognosis for patients with a variety of human cancers [8,9]. Src activation, detected by an increase in tyrosine kinase activity, has been identified in a variety of cancers, including NSCLC [10,11]. Src mediates numerous signaling pathways vital to the governance of cell transformation and homeostasis [12]. In tumor cells, the association of Src with abnormal receptor tyrosine kinases increases the tyrosine kinase activity of Src, thereby activating pro-survival pathways through PI3K/AKT, the angiogenic pathway through signal transducer and activator of transcription 3 (STAT3), the proliferation pathway through MEK/ERK, and invasion through FAK/paxillin/p130CAS [13]. In addition, Src has been reported to interact with EGFR; these proteins phosphorylate each other, and cellular Src and EGFR collaborate in cancer progression. For example, Src mediates the phosphorylation of EGFR Tyr845 to regulate survival pathways [14,15]. Mutations in EGFR and its related family members lead to functional abnormalities and, as a result, enhanced Src activation [16]. In addition, Src kinase inhibitors affect the downstream signaling cascade of Src and the inhibition of EGFR activity [17]. Preclinical studies with pharmacological Src inhibitors (dasatinib, saracatinib, and bosutinib) have provided evidence that support a role for Src as a therapeutic target in lung cancer [18,19].

Cardiac glycosides are a large family of chemical compounds that are found as secondary metabolites in several plants and in some animals. Digoxin, one of the well-known cardiac glycosides, has been approved by regulatory authorities and is widely used in the treatment of cardiac failure. Its cardiac effects are mediated through the inhibition of Na+/K+ ATPase, which leads to increased intracellular calcium concentrations and increased cardiac contractility [20]. Recently, several studies have reported that cardiac glycosides selectively inhibit proliferation and induce apoptosis and autophagy in cancer cells but not normal cells [21,22]. These results also suggested that cardiac glycoside drugs could have utility in anticancer therapy. However, the anticancer effects and molecular mechanisms of cardiac glycosides in lung cancer cells are still largely unknown. In prior, unpublished data, we identified digoxin from a small set of natural compounds as a potential candidate for reducing Src activity using an ELISA approach. In this report, we further reveal the novel mechanism of digoxin in inhibiting NSCLC malignancy, which may be through multiple Src-based signaling pathways.

## Materials and Methods

### Cell culture and drug treatment

The human lung adenocarcinoma cell lines, A549 (ATCC CCL-185), H3255 (ATCC CRL-2882), H1975 (ATCC CRL-5908), PC9 and PC9/gef [20] were cultured at 37°C in a humidified atmosphere of 5% CO_2_. Cells were maintained in RPMI 1640 (GIBCO BRL, Grand Island, NY, USA) with 10% heat-inactivated fetal bovine serum (GIBCO BRL), and 1% penicillin and streptomycin (GIBCO BRL). Digoxin was purchased from Sigma-Aldrich Chemical Company (St. Louis, MO, USA.) and prepared at a concentration of 100 mM in dimethyl sulfoxide (DMSO) as a stock solution. The working solution was freshly prepared by dilution with media to the desired concentrations. The vehicle control was 0.1% DMSO.

### Transfection

A549 cells were seeded in 6 cm dishes at 5×10^5^ cells/dish or in 96 wells at 5×10^3^ cells/well and transfected with pEGFP-N3-Src Y527F or pEGFP-N3 empty vector (Clontech, Mountair View, CA, USA) using Lipofectamine reagent (Invitrogen), according to the manufacturer’s protocol.

### Western blot analysis

Western blot analysis was performed as described previously [21]. The primary antibodies used for Western blot analyses included anti-phospho-Src (Tyr418) (Invitrogen, Carlsbad, CA, USA), anti-phospho-FAK (Tyr576) (Invitrogen), anti-FAK (Invitrogen), anti-GAPDH (Invitrogen), anti-phospho-EGFR (Tyr1068) (Cell Signaling, Beverly, MA, USA), anti-phospho-STAT3 (Tyr705) (Cell Signaling), anti-phospho-PI3K (Tyr458) (Cell Signaling), anti-AKT (Cell Signaling), anti-phospho-SAPK/JNK (Thr183/Tyr185) (Cell Signaling), anti-SAPK/JNK (Cell Signaling), anti-phospho-Paxillin (Tyr118) (Cell Signaling), anti phosphor-p130Cas (Tyr410) (Cell Signaling), anti-EGFR (Santa Cruz Biotechnology, Santa Cruz, CA, USA), anti-STAT3 (Santa Cruz Biotechnology), anti-PI3K (Santa Cruz Biotechnology), anti-phospho-MEK1/2 (Ser218/Ser222) (Santa Cruz Biotechnology), anti-MEK (Santa Cruz Biotechnology), anti-phospho-ERK (Tyr204) (Santa Cruz Biotechnology), anti-ERK2 (Santa Cruz Biotechnology), anti-Paxillin (Santa Cruz Biotechnology), anti-p130 Cas (Santa Cruz Biotechnology), anti-phospho-AKT (Ser473) (Millipore, Billerica, MA, USA)

### Real-time reverse transcription PCR

The Src, EGFR, STAT3, and FAK mRNA levels were detected with SYBR Green real-time RT-PCR on an ABI Prism 7300 sequence detection system (Applied Biosystems, Foster, CA, USA). TATA-box binding protein (TBP) was used as an internal control (GenBank X54993). All details of the empirical procedures and calculations have been described previously [21]. The following primers were used: Src forward, 5’-GAGGCCCAGGTCATGAAGAA-3’; Src reverse, 5’-CCCTTGAGAAAGTCCAGCAAA-3’; EGFR forward, 5’-CTGGCAGCCAGGAACGTACT-3’; EGFR reverse, 5’-GCCATCCACTTGATAGGCACTT-3’; STAT3 forward, 5’-CCCTTTGGAACGAAGGGTACA-3’; STAT3 reverse, 5’-AAGTGACGCCTCCTTCTTTGC-3’; FAK forward, 5’- GAGAGCTGAGGTCATTTTTGCA -3’; FAK reverse, 5’- GCAGCAATGTCCCTGTGTACA -3’; TBP forward, 5’-CACGAACCACGGGACTGATT-3’; and, TBP reverse, 5’-TTT TCTTGCTGCCAGTCTGGAC- 3’. All experiments were performed in triplicate.

### Cell viability assay

PrestoBlue Cell Viability reagent (Invitrogen, USA) was used to evaluate cell survival after digoxin treatment according to the manufacturer’s protocol. The OD readings measured at 570/600 nm by a Victor^3^ spectrophotometer (Perkin-Elmer, Boston, MA, USA) were recorded after adding the reagent and incubation. All samples were tested in triplicate.

### Colony formation assay

The detailed procedures of colony formation assay were described previously [22]. Briefly, for the anchorage-dependent growth assay, 500 cells were resuspended in RPMI and seeded in six-well plates. After 10 days, the cells were washed and fixed with 3.7% paraformaldehyde. Next, the cells were stained with 0.05% crystal violet. In contrast, for the anchorage-independent growth assay, the six-well plates were precoated with 0.7% LMP agarose in RPMI medium with 10% FBS. 1000 cells were seeded in 0.35% LMP agarose/RPMI medium with 10% FBS. After solidification, the cells were treated with digoxin for 2 weeks. The plates were stained with 0.5 mg/ml p-iodonitrotetrazolium violet. Colonies with a diameter greater than 1 mm were counted. Triplicate samples were used in the experiment.

### Invasion and migration assays

Cells were treated with various concentrations of digoxin for 24 h and seeded in transwell chambers (8-μm pore size, 6.5-mm diameter; Corning Costar Corporation, MA, USA), which were or were not coated with Matrigel (R&D Systems, Wiesbaden, Germany), as previously described [23]. The upper wells were filled with serum-free media and A549 cells (2×10^4^ or 1×10^4^ cells per well). The lower wells of the transwells contained the same media with 10% FBS and digoxin at various concentrations. The number of cells attached to the lower surface of the polycarbonate filter was counted at 400× magnification under a light microscope. All experiments were performed in triplicate.

### Statistical analysis

Results are presented as mean ± standard deviation. All experiments were performed in triplicate, and analyzed for significant differences using analysis of variance (ANOVA). *P*<0.05 was considered statistically significant.

## Results

### Promotion of NSCLC cell death by digoxin

**Fig 1:**
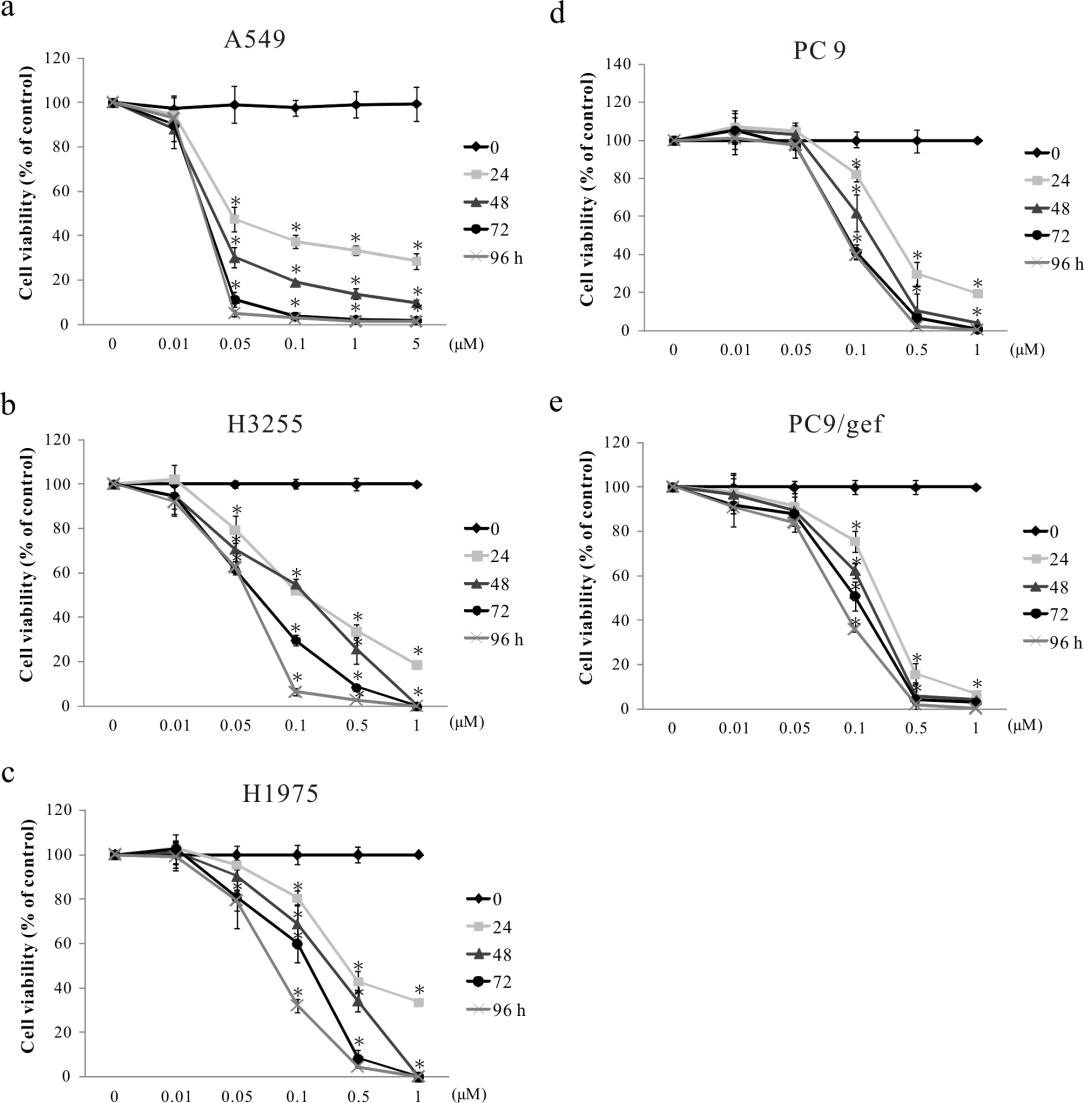
Digoxin inhibits the viability of various types of lung cancer cells. Five lung cancer cell lines, (a) A549, (b) H3255, (c) H1975, (d) PC9 and (e) PC9/gef, were treated with 6 different doses (0, 0.01, 0.05, 0.1, 1 and 5 μM) of digoxin at different time points (0, 24, 48, 72 and 96 h), and cell viability was measured using the PrestoBlue Cell Viability reagent after treatment. Quantitative data are presented as the mean±SD (n = 3); *p<0.05 compared with solvent control (0.1% DMSO) at the corresponding time point.

Digoxin, a cardiac glycoside, has been reported to have numerous anticancer effects [24,25]. To determine whether digoxin had similar cytotoxic effects in different lung cancer cell lines, five cell lines, A549, H3255, H1975, PC9 and PC9/gef, were treated with 0.01, 0.05, 0.1, 0.5 and 1 μM of digoxin for 24–96 h. PC9 cells expressing a mutant EGFR with a deletion in exon 19 are a gefitinib-sensitive NSCLC cell line. PC9/gef cells were selected from parental PC9 cells that had been continuously exposed to increasing concentrations of gefitinib [23]. The results showed that cell viability was affected in a dose- and time-dependent manner (Fig 1A–1E). The greatest effects were noted in A549 cells (IC_50_ = 0.048, 0.036, 0.030 and 0.029 μM for 24, 48, 72 and 96 h, respectively), and the second greatest effects were noted in H3255 cells (IC_50_ = 0.104, 0.107, 0.070 and 0.057 μM for 24, 48, 72 and 96 h, respectively). After 72 h of exposure to the drug, the IC_50_ values for digoxin in PC-9 and PC-9-IR cells were 0.0917 and 0.101 μM, respectively.

### The role of digoxin in the anticancer effects

**Fig 2:**
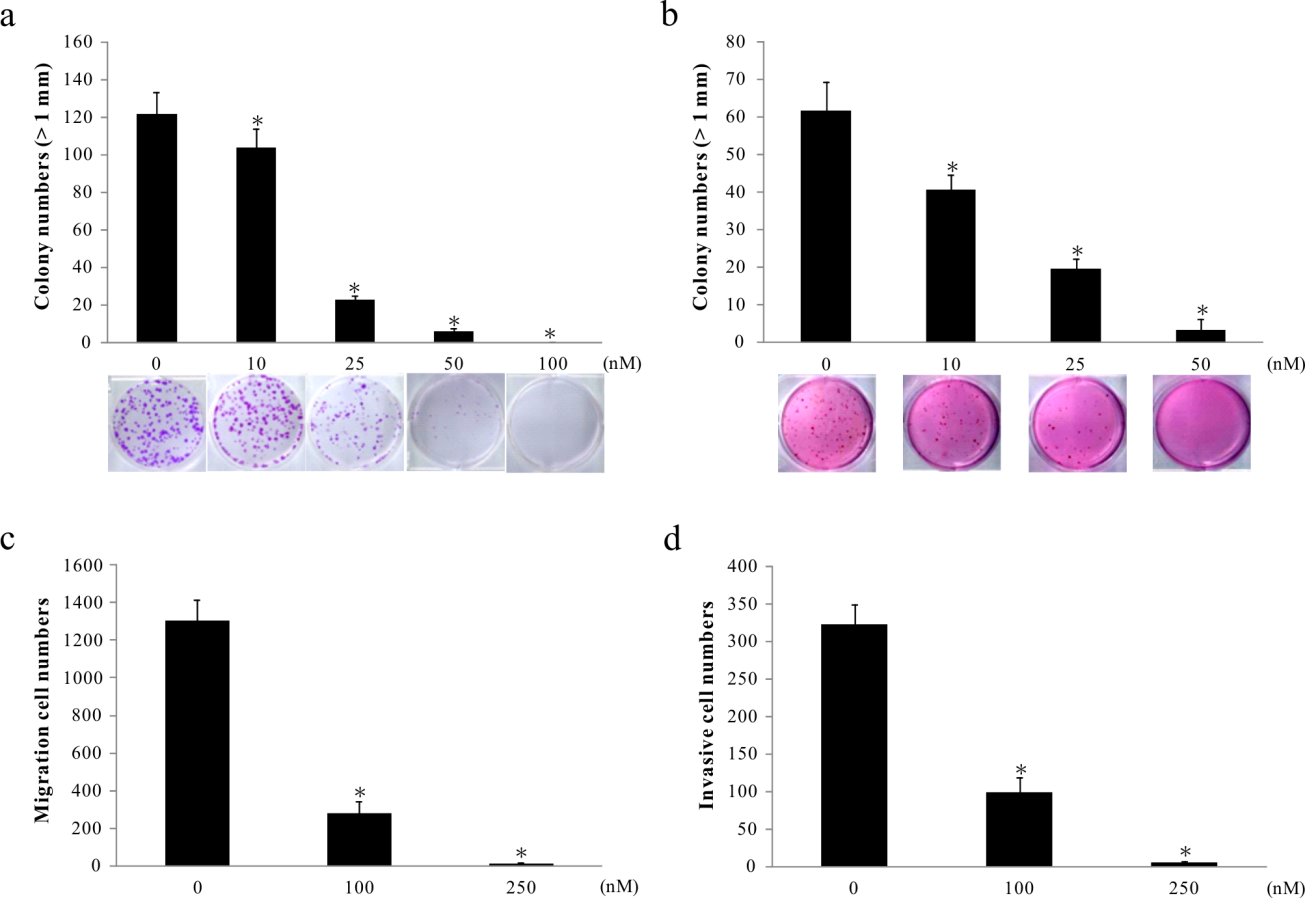
Suppression of in vitro colony formation, migration and invasion capabilities of A549 cells by digoxin treatment. A549 cells grown in a culture dish with (b) or without (a) soft agar were treated with the desired concentrations of digoxin and then subjected to colony formation analyses. (c) Digoxin decreases A549 cell migration ability, as assessed by the transwell migration assay. (d) Invasiveness of A549 cells treated with digoxin was evaluated using a Matrigel-based transwell invasion assay. *p<0.05 compared with vehicle-treated control (0.1% DMSO). Each treatment was independently performed in triplicate.

To investigate the anticancer effects of digoxin, we next performed cell anchorage-dependence, anchorage-independence, migration and invasion assays. Our results demonstrated that digoxin inhibited the formation of A549 cell colonies in a dose-dependent manner (10, 25, 50 and 100 nM) within weeks, irrespective of anchorage-dependent (Fig 2A) or anchorage-independent growth (Fig 2B). To further evaluate the antitumor effects of digoxin on migration and invasion, A549 cells were pre-treated with varying concentrations of digoxin for 24 h and then subjected to invasion and migration assays for 12 h and 16 h, respectively. Our data demonstrated that digoxin significantly inhibited cell migration and invasion at 100 nM compared with solvent control (0.1% DMSO, Fig 2C and 2D).

### Digoxin inhibits the activation of Src and related proteins

**Fig 3:**
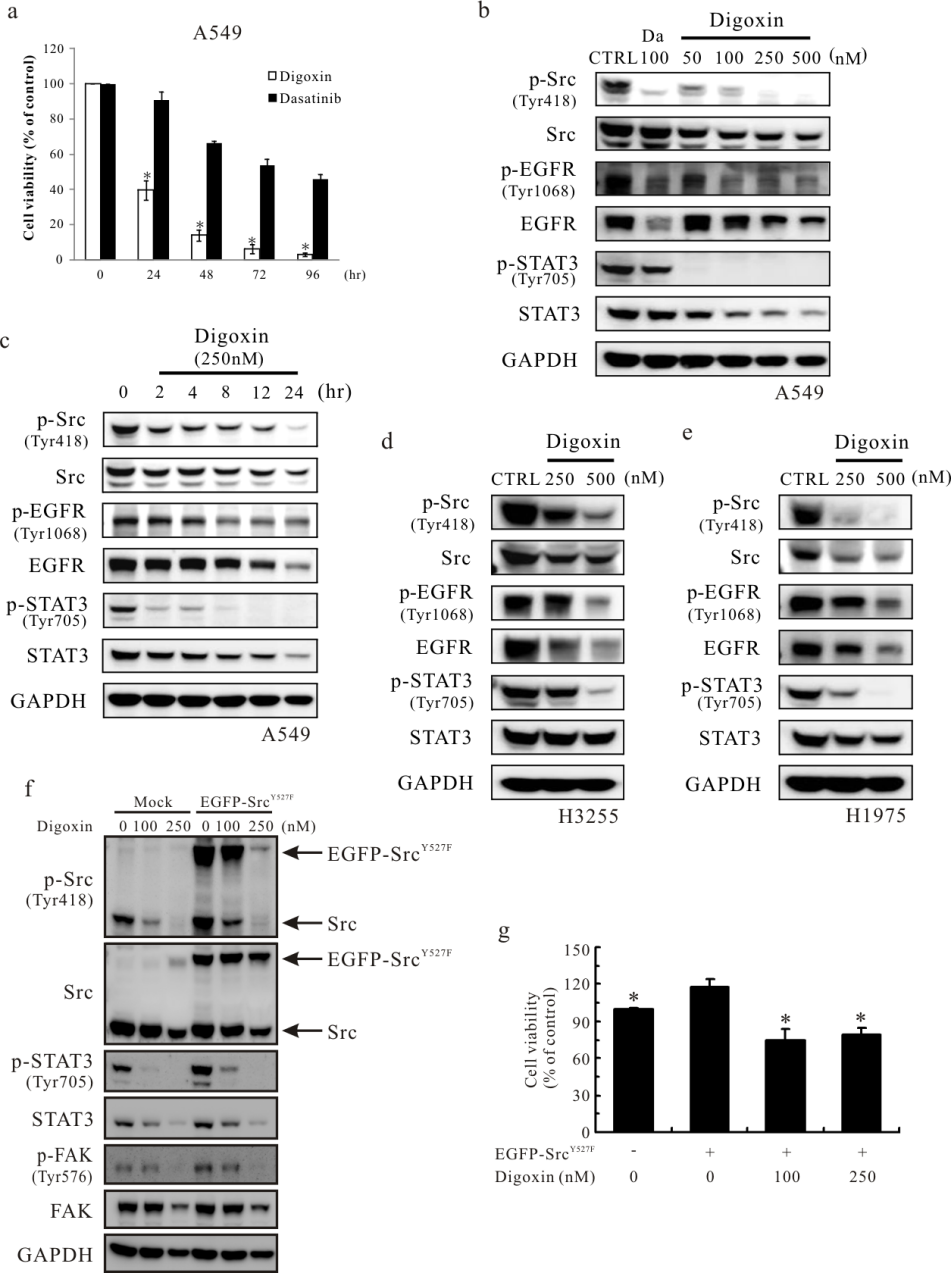
Digoxin inhibits phosphorylation of Src and the related EGFR/STAT3 pathway in various types of lung cancer cells in a dose- and time-dependent manner. (a) A549 cell viability was measured using the Prestoblue Cell Viability Reagent at different time points (0, 24, 48, 72 and 96 h) with or without 100 nM digoxin or dasatinib. (b) Analysis of phosphorylated and non-phosphorylated Src, EGFR and STAT3 in A549 cells in a dose-dependent manner. The cancer cells treated with or without various concentrations of digoxin (50, 100, 250 and 500 nM) or 100 nM dasatinib for 24 h were analyzed by Western blot. (c) Analysis of phosphorylated and non-phosphorylated Src, EGFR and STAT3 in A549 cells in a time-dependent manner. After digoxin treatment (250 nM) for 2, 4, 8, 12 or 24 h, the cancer cells were harvested and analyzed by Western blot. The effect of digoxin on Src, EGFR, and STAT3 in (d) H3255 cells and (e) H1975 cells. GAPDH was used as an internal control. Each treatment was independently performed in triplicate. (f) Effect of Src Y527F on the phosphorylation of STAT3 and FAK after digoxin treatment in A549 cells. Mock and EGFP-Src Y527F transfectants were treated with 100 or 250 nM of digoxin for 24 h. The phosphorylation and total protein levels of Src, STAT3 and FAK were detected by Western blotting. GAPDH was used as an internal control. Each treatment was independently performed in triplicate. (g) Digoxin inhibits the viability of cells transfected with the Src Y527F mutant. After transfection with Src Y527F and treatment with 100 or 250 nM of digoxin for 24 h, A549 cell viability was determined by PrestoBlue Cell Viability reagent. Quantitative data are presented as the mean±SD (n = 3); *p<0.05 compared with the vehicle control (0.1% DMSO) of EGFP-Src Y527F transfectant.

Dasatinib (BMS-354825), a Src kinase inhibitor (SKI), has been identified as an efficient target therapy drug in vitro and in clinical research [26,27]. In this study, dasatinib was used as a positive control. A cytotoxicity assay indicated that cell viability was significantly decreased by digoxin, and the effects were stronger than those of dasatinib at the same concentration (100 nM) for 24, 48, 72 and 96 h (Fig 3A). To determine whether digoxin affects the phosphorylation of Src and related proteins in a dose-dependent manner, a Western blot assay was performed. The results indicated that the phosphorylation of Src Y418, EGFR Y1068 and STAT3 Y705 was reduced in a dose-dependent manner (50–500 nM) by digoxin in the A549 lung cancer cell line that possesses wild-type EGFR (Fig 3B). We also determined that digoxin reduced the phosphorylation of Src Y418, EGFR Y1068 and STAT3 Y705 in a time-dependent manner from 2 to 24 h in A549 cells (Fig 3C). Similar results were obtained in other lung cancer cell lines, e.g., the H3255 cell line bearing an L858R EGFR mutant and the H1975 cell line containing an L858R/T790M EGFR mutant (Fig 3D and 3E). To confirm that the effect of digoxin is via regulation of Src activity, a constitutively activated Src mutant construct (Src Y527F) [28] was used. The results showed that overexpression of Src Y527F enhanced the phosphorylation of STAT3 and FAK. However, the phosphorylation of Src Y418, STAT3 Y705 and FAK Y576 was still reduced in digoxin-treated cells (Fig 3F). To further evaluate the effects of Src Y527F on cell viability, A549 cells were transfected with Src Y527F for 18 h and then treated with 100 or 250 nM of digoxin for 24 h. Mutant Src Y527F-transfected cells showed an increase of cell viability, compared to mock transfectants (α = 0.05, p<0.05). The data also showed that 100 and 250 nM digoxin significantly inhibited cell viability in Src Y527-transfected cells compared with the vehicle control (0.1% DMSO) of Src Y527 transfectant (α = 0.05, p<0.05) (Fig 3G). These results indicated that digoxin can cause cytotoxic effect and reduce the phosphorylation of Src, STAT3 and FAK in cells with constitutive Src activation.

### Digoxin inhibits Src activity and downstream signaling

**Fig 4:**
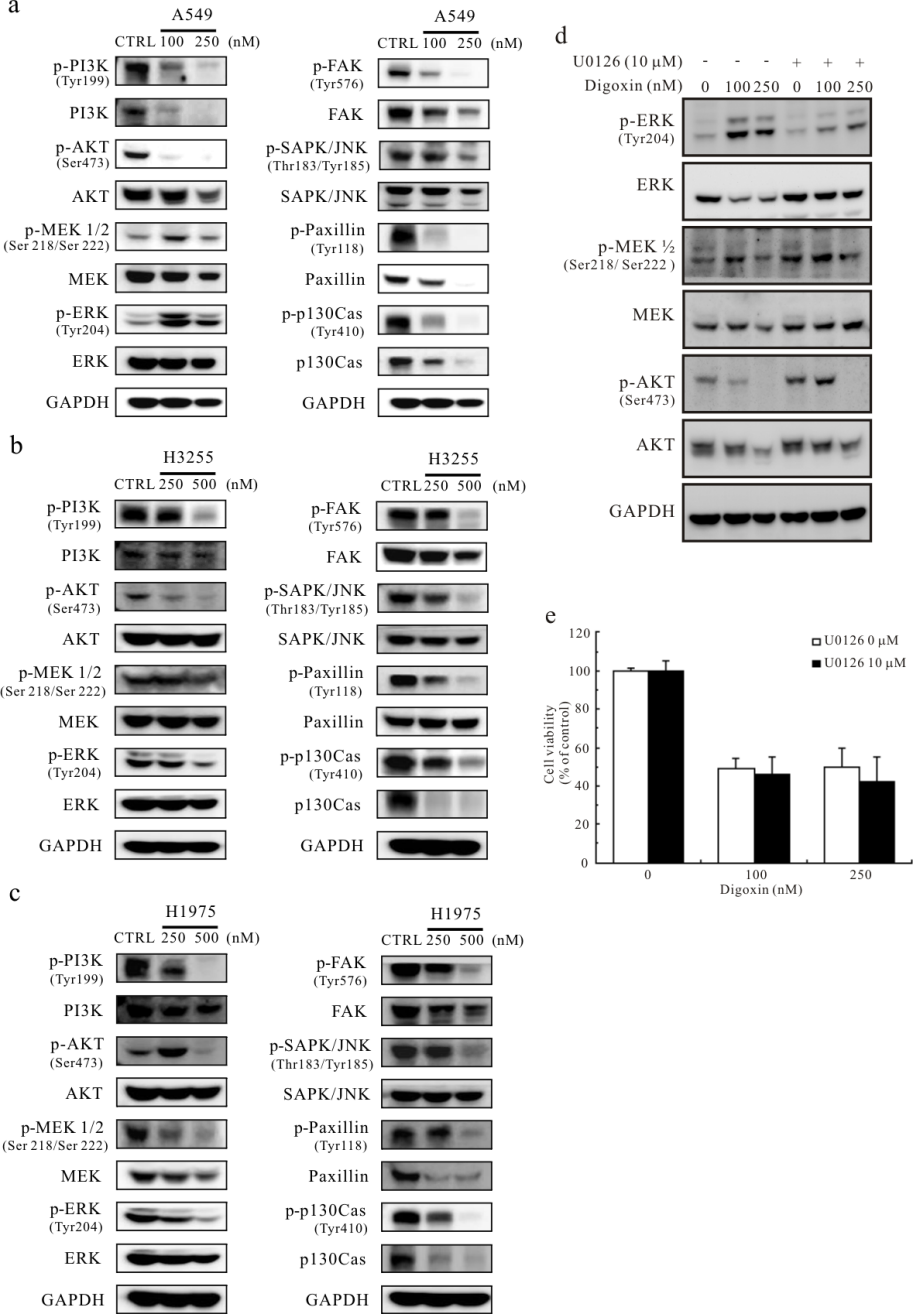
The effect of digoxin on cell signaling. Western blotting analyses of phosphorylated and non-phosphorylated PI3K, AKT, MEK1/2, ERK, FAK, SAPK/JNK, paxillin and p130Cas in (a) A549, (b) H3255, and (c) H1975 cell lines after a 24-h treatment with 100, 250 or 500 nM digoxin. The solvent control was 0.1% DMSO; GAPDH was used as an internal control. (d) Western blotting analyses of phosphorylated and total ERK, MEK1/2 and AKT in A549 cells after a 24-h treatment with 10 μM of U0126 and 100 or 250 nM of digoxin. GAPDH was used as an internal control. (e) ERK inhibitor has no effect on digoxin-induced cell death. A549 cells were treated with the designated concentrations of U0126 and digoxin. The cell viability was measured by PrestoBlue Cell Viability reagent. Data are presented as means ± S.D. (n = 3).

The effects of digoxin on Src downstream targets were detected by Western blotting in digoxin-treated cell lines (Fig 4A–4C). A significant inhibition of PI3K, FAK, SAPK/JNK, paxillin and p130Cas activities by digoxin was shown in three cell lines at 250/500 nM and below. In particular, A549 cells, the most sensitive to digoxin growth inhibition, showed the promotion of p-MEK1/2 and p-ERK by digoxin at 100 nM (Fig 4A). H3255 cells were the second most sensitive to digoxin because p-Src was inhibited (Fig 3D). In this cell line, digoxin slightly reduced p-MEK1/2 and p-ERK at 500 nM (Fig 4B). In addition, even though digoxin significantly inhibited the expression of p-Src at 250 nM in H1975 cells (Fig 3E), an increase in p-AKT activity was in concert with the inhibition of p-MEK and p-ERK (Fig 4C). To investigate the role of ERK1/2 in the effect of digoxin, A549 cells were treated with the ERK inhibitor U0126 and 100 or 250 nM of digoxin for 24 h. The cells were then subjected to Western blotting (Fig 4D) and cell viability (Fig 4E) assay. The results showed that inhibition of ERK1/2 phosphorylation was unable to prevent the cytotoxic effects of digoxin or the promotion of p-ERK and p-MEK1/2 by digoxin at 100 nM. This result suggested that digoxin may affect other signaling pathways and inhibit other growth factors or protein kinases to regulate cell growth in these cell lines.

### Digoxin reduces mRNA expression of Src and related proteins

**Fig 5:**
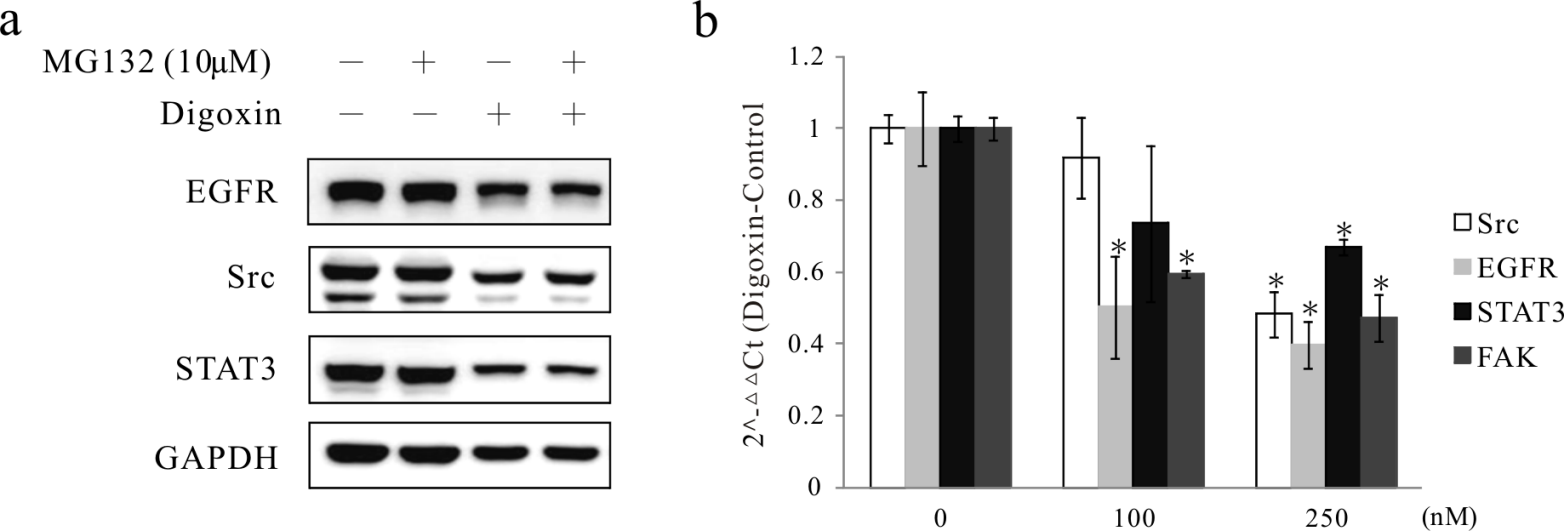
Inhibition of mRNA expression of Src, EGFR, STAT3 and FAK in digoxin-treated cells. (a) A549 cells were pretreated with or without MG132 (10 μM) for 2 h, then treated with digoxin (250 nM) or 0.1% DMSO (solvent control) and collected at 24 h. Protein expression was evaluated by Western blot analysis. GAPDH was a control for protein loading and transfer. (b) Cells were treated with digoxin (100 and 250 nM) for 24 h, and the detection of Src, EGFR, STAT3 and FAK mRNA expression was determined by real-time RT-PCR. *p<0.05, significantly different from the vehicle-treated control (0.1% DMSO). Each experiment was independently performed triplicate.

Figs 3 and 4 showed that digoxin not only inhibited Src and related protein kinase activity but also reduced the quantity of Src and related protein kinases. We further scrutinized whether this effect may arise from protein instability or transcriptional down-regulation. A549 cells were pretreated with the proteasome inhibitor MG-132 at 10 μM for 2 h before exposure to 250 nM digoxin for another 24 h. However, in the presence of the proteasome inhibitor, the expression of digoxin-reduced Src, EGFR and STAT3 was not altered significantly compared with that in the cells treated with digoxin alone (Fig 5A). We further xamined whether digoxin decreased the expression of Src, EGFR, STAT3, and FAK mRNAs in A549 cells using reverse transcription quantitative polymerase chain reaction (RT-qPCR; Fig 5B). We observed a down-regulation of EGFR and FAK mRNA expression to 0.5- and 0.6-fold, respectively, at 100 nM digoxin in contrast to solvent control (p<0.05) and down-regulation of Src and STAT3 mRNA expression to 0.48- and 0.67-fold, respectively, at 250 nM digoxin (p<0.05). These results indicated that the expression of Src and related proteins is regulated by digoxin, which, in turn, impacts transcriptional regulation.

## Discussion

Lung cancer has a high mortality rate and is one of the most common malignant tumors. Recent successes in targeted therapy have improved the outcomes of cancer treatment. A regimen of EGFR TKIs, including gefitinib and erlotinib, is the standard first-line treatment against advanced NSCLC in patients harboring activating EGFR mutations. [29]. However, this approach inevitably promotes TKI resistance. Src, a well-known oncogene, plays an important role in many signaling pathways and maintains activation in various cancers, including lung cancer [30]. In this report, we found that digoxin significantly inhibits Src phosphorylation in NSCLC cells with varying EGFR genotypes, including wild type, an L858R mutant, and an L858R/T790M mutant. Furthermore, we revealed the ability of digoxin to inhibit lung cancer proliferation, migration, and invasion in vitro. In addition, we show a multi-functional role for digoxin possibly involving signaling pathways, which to our knowledge, has not been previously reported.

Digoxin is a natural compound extracted from foxglove (*Digitalis purpurea* L.) [31], which belongs to a group of cardiac glycosides that can bind and inhibit sodium pumps. It has been used clinically to treat heart failure and atrial arrhythmia via inhibition of Na^+^/K^+^-ATPase for many years [32], and it significantly inhibits the growth of pancreatic cancer cells [33]. As previously noted, low concentrations of cardiac glycosides have been shown to have low cytotoxicity in normal cells [32]. In addition, digoxin has been shown to inhibit primary tumor growth and the metastasis of cancer cells from breast to lung in an orthotopic model in which human breast cancer cells were implanted into SCID mice [34]. At the molecular level, digoxin down-regulates NDRG1 and VEGF through the inhibition of HIF-1α under hypoxic conditions in A549 cells [35] and induces autophagy to account for the growth inhibitory effects in NSCLC cells through the regulation of mTOR and ERK1/2 signaling pathways [36]. In this study, we found that digoxin reduced the phosphorylation of Src in a dose (50–500 nM) and time (2–24 h)-dependent manner. Our data also revealed that digoxin reduced the phosphorylation of exogenous, constitutively active Src in transfected cells. However, a previous report indicated that digoxin slightly elevated the phosphorylation of both Src and ERK in NSCLC cells within 10 min at a concentration of 100 nM and ultimately resulted in the reduction of p53 protein synthesis [37]. We speculate that the discrepancy may be due to the exposure time to digoxin. However, this is the first study to demonstrate that digoxin can inhibit not only Src but also the phosphorylation and expression of EGFR and STAT3 to further retard cancer cell proliferation, migration and invasion. Our results support the potential use of digoxin as a multi-target agent in cancer therapies.

TKIs with activities against EGFR are effective in lung cancer patients with EGFR mutations; however, resistance emerges over time. Thus, finding a new agent against EGFR wild-type and TKI-resistant lung cancers is a necessity. In our study, an EGFR wild-type lung cancer cell line, A549, was used to screen Src kinase inhibitors. NSCLC patients with an Arg substitution for Leu at position 858 or a deletion of exon 19 in EGFR are responsive to EGFR TKIs [38]. Several clinical studies have indicated that the amplification of a T790M mutation in EGFR contributes to the acquisition of EGFR TKI resistance [39]. Therefore, TKI-sensitive lung cancer cell lines (PC9, exon 19 deletion; H3255, L858R EGFR mutant) [40] and TKI-resistant lung cancer cell lines (PC9/gef, exon 19 deletion, acquired resistance; H1975, L858R/T790M EGFR mutant) [38] were used to evaluate the anticancer effectiveness of digoxin. Our results indicated that A549 cells were the most sensitive to digoxin at low concentrations, whereas other lung cancer cells responded to digoxin at concentrations ranging from 250–500 nM.

A recent study indicated that Src activation governs a variety of pathways, including survival, angiogenesis, proliferation, migration, and invasion, through a variety of proteins, including PI3K/AKT, STAT3, MEK/ERK and FAK/paxillin/p130CAS [13]. Liu et al. demonstrated that cell proliferation is regulated by PI3K/AKT and RAF/MEK/ERK signaling pathways [41]. In a separate study, ERK1/2 activation was simultaneously found in digoxin-induced autophagy [36]. In the present study, digoxin reduced the phosphorylation of PI3K and ERK in various types of lung cancers at 250 nM but promoted the phosphorylation of MEK and ERK at a low concentration (100 nM) in A549 cells. However, inhibition of the ERK pathway did not alter the effects of digoxin, indicating that there might be other pathways involved in the effects of digoxin. We suggest that digoxin may inhibit cancer cell growth through inhibition of the PI3K/AKT signaling pathways, leading to autophagy at different concentrations. STAT3 is activated in EGFR wild-type NSCLC and correlates with cancer progression, including cell survival, migration and invasion [42]. Sorafenib and one of its derivatives (SC-1) have been reported to inhibit EGFR wild-type NSCLC growth and induce apoptosis via the SHP-1/STAT3 pathway [43]. In this study, we found that digoxin not only reduced STAT3 phosphorylation in EGFR wild-type NSCLC but also in EGFR mutant-type NSCLC. Additionally, activation of FAK-Src molecular scaffolds and p130Cas-JNK signaling cascades by α1-integrins promotes the invasion of colon cancer cells [44]. Furthermore, paxillin is aberrantly regulated in various malignancies and involved in tumor growth and invasion. The ectopic expression of paxillin facilitates cell proliferation and migration, whereas paxillin knockdown inhibits these events in gastric cells [45]. Our data indicated that digoxin suppresses FAK-Src, paxillin, PI3K/AKT, MEK/ERK, STAT3 and p130Cas-JNK signaling pathways in various EGFR-containing lung cancer cells, suggesting that digoxin inhibits cancer cell invasion by decreasing Src activation and the activation of related signaling pathways.

**Fig 6:**
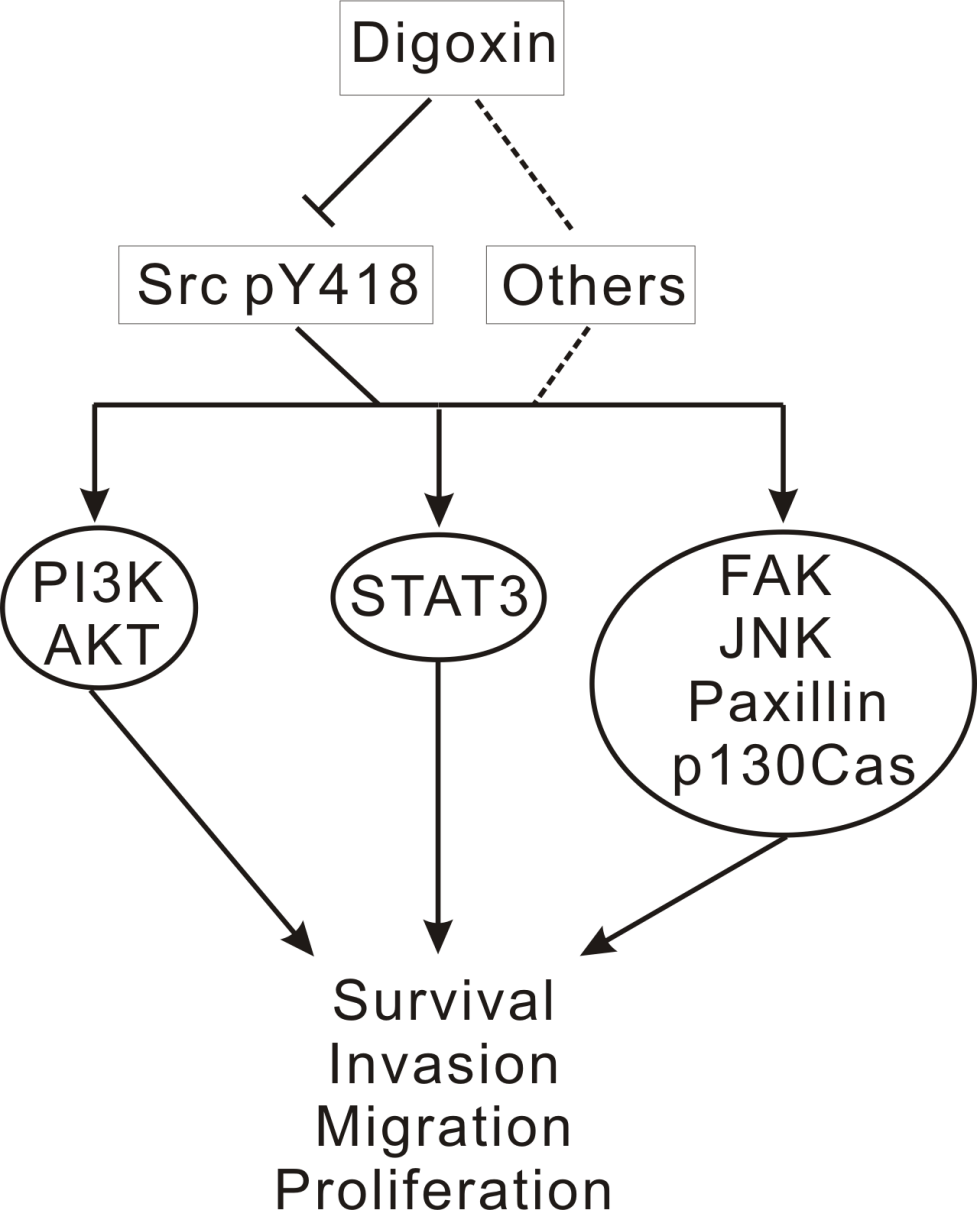
A proposed model of digoxin-mediated anticancer function. In digoxin-treated cells, the phosphorylation of Src and its related proteins was inhibited, which may lead to the inhibition of lung cancer cell proliferation, migration, and invasion.

In conclusion, using in vitro drug screening and cell function assays, we discovered that digoxin may suppress lung cancer progression by inhibiting Src activation and the activation of related pathways, including cell proliferation, migration, and invasion. However, we still cannot exclude the possibility that other signaling pathways and proteins play some roles in digoxin-induced effects (Fig 6). Taken together, we propose that digoxin will be important in anticancer drug development.
